# Amphetamin induzierte zerebrale Vaskulitis – ein Fallbericht

**DOI:** 10.1007/s00115-021-01125-w

**Published:** 2021-04-29

**Authors:** Jonathan Vöglein, Johannes Levin, Robert Forbrig, Thomas Liebig, Marianne Dieterich, Adrian Danek

**Affiliations:** 1 Neurologische Klinik und Poliklinik, LMU Klinikum, München, Deutschland; 2 Deutsches Zentrum für Neurodegenerative Erkrankungen (DZNE), München, Deutschland; 3 Munich Cluster for Systems Neurology (SyNergy), München, Deutschland; 4 Institut für diagnostische und interventionelle Neuroradiologie, LMU Klinikum, München, Deutschland; 5 Deutsches Schwindel- und Gleichgewichtszentrum (DSGZ), LMU Klinikum, München, Deutschland

## Hintergrund

Zerebrale Vaskulitiden sind eine wichtige Differenzialdiagnose juveniler Schlaganfälle. Häufigste Ursachen sind primäre ZNS(zentrales Nervensystem)-Vaskulitiden und ZNS-Beteiligungen bei systemischer Vaskulitis. Weiter kommen eine infektiöse oder parainfektiöse Genese vor, eine Beteiligung im Rahmen einer (z. B. rheumatischen) Systemerkrankung, eine paraneoplastische Genese sowie eine medikamenten- oder drogeninduzierte Vaskulitis. Relevante Differenzialdiagnosen sind nichtinflammatorische Vaskulopathien wie z. B. bei Arteriosklerose, reversiblem zerebralem Vasokonstriktionssyndrom (RCVS) und idiopathischer Moyamoya-Erkrankung. Behandlung und Prognose zerebraler Vaskulitiden werden von der Ursache bestimmt [2, 3].

## Kasuistik

**Figure 1:**
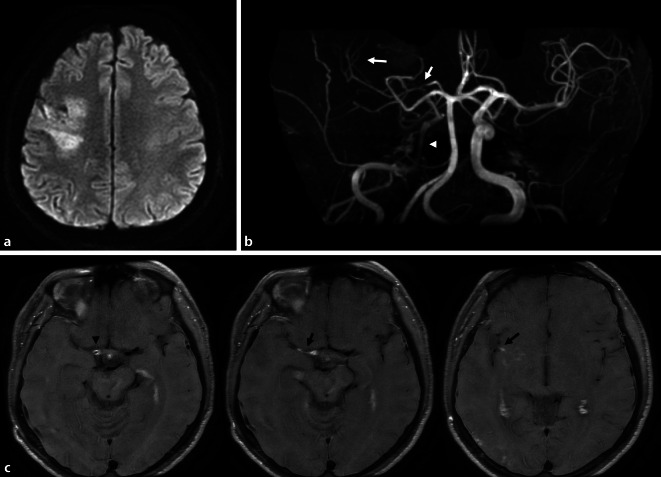

Der 35-jährige Küchenhelfer stellte sich mit einer subakuten Parese der linken oberen Extremität vor. Korrelat in der zerebralen MRT war ein Mediateilinfarkt rechts (Abb. 1a). Anamnestisch berichtete der Patient von seit 4 Wochen bestehenden fluktuierenden, pochenden Kopfschmerzen rechts (NAS 8/10). Ein Trauma oder kardiovaskuläre Risikofaktoren waren in der Vorgeschichte nicht zu erfragen. Zwei Jahre zuvor war der Patient an einer atraumatischen Femurkopfnekrose rechts unklarer Ursache erkrankt, die operativ behandelt wurde. In der Familienanamnese wurden keine Schlaganfälle berichtet. Eine relevante Dauermedikation bestand nicht, aber auf Nachfrage wurde ein Amphetaminkonsum seit 2012, zuletzt vor 2 Wochen, berichtet.

In ergänzenden MRT-Sequenzen zeigten sich im Seitenvergleich rechts ein verringertes Arterienkaliber bzw. eine fehlende Abgrenzbarkeit in der flussbasierten Darstellung der parakavernosalen und terminalen ACI sowie der gesamten A. cerebri media (Abb. 1b). In der „Black-blood“-Sequenz, welche fließendes Blut durch Signalunterdrückung auch nach Kontrastmittelgabe hypointens zur Darstellung kommen lässt und deshalb besonders zur Darstellung pathologischer und segmental gesteigerter Kontrastmittelanreicherungen der Gefäßwand geeignet ist [9], stellten sich verdickte, kontrastmittelanreichernde Gefäßwände von der rechten distalen Arteria carotis interna bis in die Mediaäste der Inselregion (M2) reichend dar (Abb. 1c).

Die Liquoruntersuchung ergab unauffällige Befunde hinsichtlich der Routineparameter, der Proteindifferenzierung, der oligoklonalen Banden und der Erregerdiagnostik. Die differenzialdiagnostische Aufarbeitung inklusive Ganzkörper FDG-PET-CT zur Abklärung eines potenziellen Gefäßbefalls im Rahmen einer systemischen Vaskulitis und zur Suche nach einer neoplastischen Erkrankung, die ein paraneoplastisches Syndrom verursachen könnte, Laboruntersuchungen (Elektrolyte, Nierenwerte, Leberwerte, Cholestaseparameter, CRP, Pankreaswerte, LDH, CK, BSG, Immunelektrophorese, Serumgesamteiweiß, Harnsäure, Differenzialblutbild, Erythrozytenindices, Hämoglobin, Hämatokrit, Gerinnungsdiagnostik, PSA, Kryoglobuline, Komplementfaktoren, Rheumafaktor, ANA, ENA, ANCA, Cardiolipin-Antikörper, β‑2-Glykoprotein-Antikörper, TSH, Urinstix und -sediment) und organspezifischer Diagnostik (Gastroskopie, Koloskopie, Lungenfunktionsuntersuchung, Ultraschalluntersuchung der Beinvenen, HNO-, gastroenterologische, orthopädische, dermatologische und ophthalmologische Untersuchung) zeigte keine Hinweise auf eine systemische Vaskulitis oder eine maligne Grunderkrankung. Liquor- und Blutdiagnostik lieferten keinen Anhalt für eine paraneoplastische Erkrankung. Magnetresonanztomographisch fanden sich keine weiteren Gefäß- oder Hirnparenchymläsionen, keine SAB, keine auf eine idiopathische Moyamoya-Erkrankung hinweisende Kollateralisierung und keine Dissektion. Bei Vasospasmen, z. B. im Rahmen einer SAB oder eines RCVS, findet sich in der „Black-blood“-Sequenz typischerweise keine Kontrastmittelanreicherung der Gefäßwand. Nach 2 Wochen war der Gefäßbefund magnetresonanztomographisch unverändert. Da sich in der MR-Angiographie keine Hinweise auf weitere Gefäßpathologien fanden, der Befund der „Black-blood“-Sequenz typisch für eine Entzündung der Gefäßwand war und sich ein klinisch stabiler Verlauf zeigte, wurde auf eine invasive Gefäßdiagnostik mittels digitaler Subtraktionsangiographie verzichtet. In der farbkodierten Duplexsonographie der extrakraniellen Arterien fand sich ein prästenotisches Flussprofil mit im Seitenvergleich reduzierten Flusswerten. Eine Arteriosklerose oder Stenosen der extrakraniellen Arterien lagen nicht vor. In der kardialen Diagnostik mittels transthorakaler Echokardiographie und automatisiertem Herzrhythmusmonitoring über 72 h zeigten sich keine pathologischen Befunde. Blutdruckwerte, HbA1c und LDL-Cholesterin waren unauffällig. HIV- und Hepatitis-B/C-Serologien waren negativ.

Da bezüglich der Sekundärprophylaxe für den beschriebenen Einzelfall keine gesonderte Evidenz vorlag, wurde der Patient leitliniengerecht mit 100 mg Aspirin und 40 mg Atorvastatin täglich behandelt [8]. Es erfolgten wiederholte psychotherapeutische Kurzinterventionen mit dem Ziel einer Amphetaminabstinenz [7].

Im Verlauf kam es unter Ergotherapie zu einer Besserung der Parese und nach 2 Jahren berichtete der Patient von einer Amphetaminabstinenz seit dem Schlaganfall. Anamnestisch und klinisch fanden sich keine Hinweise auf eine erneute zerebrale Ischämie.

## Diskussion

Der bildgebende Befund war ausschlaggebend für die Diagnose einer zerebralen Vaskulitis. Passend hierzu fanden sich zum Verlauf und der Lokalisation passende rechtseitige Kopfschmerzen. Die zum Zeitpunkt des Schlaganfalls 2 Jahre zurückliegende atraumatische Femurkopfnekrose, ebenfalls eine ischämische Erkrankung [1], könnte auf eine systemische Vaskulitis hindeuten. In der umfangreichen differenzialdiagnostischen Aufarbeitung fanden sich keine Hinweise auf die Ursache der zerebralen Vaskulitis, sodass der Amphetaminkonsum in Betracht kam. In der Literatur wurde von 3 Fällen mit amphetaminkonsumassoziierten ZNS-Vaskulitiden berichtet, die autoptisch gesichert wurden. Die neuropathologischen Untersuchungen zeigten nekrotisierende Gefäßwandveränderungen [4, 5]. Eine Studie mit Rhesusaffen, denen für den Missbrauch beim Menschen adaptierte Amphetamindosen verabreicht wurden, zeigte in 80 % der Primaten angiographische Veränderungen und Sektionsbefunde einer nekrotisierenden Vaskulitis [6]. Damit ist in diesem Fall eine amphetamininduzierte zerebrale Vaskulitis die wahrscheinlichste Diagnose. Die Evidenz für eine adäquate Therapie ist spärlich. De facto existiert nur ein Fallbericht einer Patientin mit bioptisch gesicherter amphetaminkonsumassoziierter nekrotisierender ZNS-Vaskulitis, in dem über eine Vollremission angiographischer Veränderungen im mittelfristigen Verlauf unter einer Behandlung mit Cyclophosphamid berichtet wurde [5]. Aufgrund des stabilen klinischen und bildgebenden Befundes sowie der atraumatischen Femurkopfnekrose in der Vorgeschichte, deren Hauptrisikofaktor eine Kortisontherapie darstellt [1], erfolgte bei dem Patienten keine immunsuppressive Behandlung. Interessant ist die Frage, warum nur ein Gefäßterritorium von der Vaskulitis betroffen war (oder zwei Gefäßterritorien, wenn die Femurkopfnekrose in der Vorgeschichte als Manifestation einer systemischen Vaskulitis gesehen wird). Dies könnte der unterschiedlichen Suszeptibilität verschiedener Gewebe bzw. Territorien für pathologische Prozesse geschuldet sein. Zum anderen könnten andere subtile Vaskulitismanifestationen der Diagnostik entgangen sein. Zusammenfassend stellt die zerebrale Vaskulitis neben anderen Auswirkungen von Amphetaminen auf das zentrale Nervensystem (Tab. 1) eine wichtige potenzielle Folge eines Amphetaminkonsums dar.

**Table 1:** 

Folge von Amphetaminkonsum	Symptome
Zerebrale Ischämie	Fokale neurologische Ausfälle
Hirnblutungen (Subarachnoidalblutung, intrazerebrale Blutung)	Donnerschlagkopfschmerz, Bewusstseinsstörung, fokale neurologische Ausfälle
Epileptische Anfälle	Motorische Entäußerungen, Bewusstseinsstörung, Zungenbiss, Einnässen
Psychische Symptome	Agitation, Stimmungsschwankungen, Aggression, psychomotorische Unruhe, Halluzinationen, paranoide Wahnvorstellungen, Panik, Abhängigkeit

## Fazit für die Praxis

- Beim Verdacht auf eine ZNS-Vaskulitis muss an eine Drogenanamnese gedacht werden.
- Amphetaminabstinenz steht therapeutisch im Vordergrund.
- Gegebenenfalls kann eine immunsuppressive Behandlung im Rahmen eines individuellen Therapiekonzeptes erfolgen.
- Die Langzeitprognose ist unklar, bei Beendigung der Amphetaminexposition möglicherweise gut.
